# From nucleotides to numbers: a comprehensive review of RNA feature extraction methods for computational modelling

**DOI:** 10.1093/bib/bbaf701

**Published:** 2025-12-31

**Authors:** Fatemeh Safari, Jai J Tree, Fatemeh Vafaee

**Affiliations:** School of Biotechnology and Biomolecular Sciences, University of New South Wales, Sydney, NSW 2052, Australia; UNSW Biomedical AI, University of New South Wales, Sydney, NSW 2052, Australia; School of Biotechnology and Biomolecular Sciences, University of New South Wales, Sydney, NSW 2052, Australia; School of Biotechnology and Biomolecular Sciences, University of New South Wales, Sydney, NSW 2052, Australia; UNSW Biomedical AI, University of New South Wales, Sydney, NSW 2052, Australia; UNSW AI Institute, University of New South Wales, Sydney, NSW 2052, Australia

**Keywords:** RNA bioinformatics, non coding RNA (ncRNA), feature extraction, machine learning, sequence representation

## Abstract

Machine learning is a powerful approach for analysing RNA sequences, particularly for understanding the function and regulation of noncoding RNAs. A critical step in this process is feature extraction, which transforms biological sequences into numerical representations that allow computational models to capture and interpret complex biological patterns. Despite its central role, the field of RNA feature extraction remains broad and fragmented, with limited standardization and accessibility hindering consistent application. In this comprehensive review, we address the fragmentation of the field by systematically organizing over 25 feature extraction strategies into sequence- and structure-based approaches. We further conduct a comparative analysis highlighting how the choice of feature sets impacts model performance, reinforcing the importance of integrated feature engineering. To facilitate practical adoption, it also provides a curated list of publicly available tools and software packages. By consolidating methodologies and resources, this work seeks to improve reproducibility, scalability, and interpretability in machine learning-driven RNA research.

## Introduction

RNA sequencing (RNA-seq) has revolutionized transcriptomics by enabling the comprehensive analysis of RNA expression across various cell types, tissues, and biological conditions [1, 2]. Beyond quantifying gene expression, RNA-seq data support diverse applications such as the discovery of novel transcripts, annotation of noncoding RNAs (ncRNAs), and exploration of transcriptomic diversity [1]. RNA molecules, including messenger RNAs (mRNAs) and various classes of noncoding RNAs such as microRNAs (miRNAs), long noncoding RNAs (lncRNAs), small RNAs (sRNAs), and circular RNAs (circRNAs), play essential roles in gene regulation, RNA processing, epigenetic control, and molecular interactions in both prokaryotic and eukaryotic organisms [3–6]. Determining the sequence and structural properties of these RNAs is therefore critical for understanding cellular behaviour, genetic regulatory regions, and identifying biomarkers or therapeutic targets [2].

With an increasing number of RNA-seq datasets, one of the key challenges is the transformation of raw sequence data into meaningful, quantifiable features suitable for computational modelling [7, 8]. Machine learning (ML) algorithms cannot interpret nucleotide sequences in their original form and therefore require the data to be converted into informative numerical representations. This transformation is achieved through feature extraction, a vital preprocessing step that encodes sequence and structural properties into numerical formats that retain relevant biological patterns while minimizing noise and redundancy [9–11]. These extracted features facilitate the development of predictive models based on machine learning, which can be applied to various domain-specific applications in molecular biology and biomedical research. These applications include but are not limited to: RNA classification (e.g. noncoding versus coding RNAs), RNA-protein and RNA–RNA interaction prediction, transcript stability analysis, prediction of subcellular localization, functional annotation, and the design of therapeutic RNAs, including small interfering RNAs (siRNAs), RNA aptamers, and CRISPR guide RNAs. The quality and consistency of the extracted features are critical to the effectiveness of these applications, as they influence model accuracy, generalizability, and interpretability [7, 8].

In parallel with traditional feature extraction methods, deep learning-based representation learning approaches have emerged as a promising direction in computational biology [12]. Representation learning aims to automatically extract meaningful features directly from raw sequence data, thereby eliminating the need for manual feature design [13]. However, despite its potential, representation learning faces challenges such as its reliance on large datasets, susceptibility to overfitting when applied to small datasets, significant computational requirements, and function as black-box models, limiting transparency in decision-making [14–17]. For small to medium-sized datasets, traditional machine learning methods such as support vector machines (SVMs), random forests, and gradient boosting remain effective alternatives. Although they require structured feature engineering, which involves additional preprocessing, this process enables a more interpretable and systematically controlled modelling approach [18].

Despite increasing interest in ML for RNA analysis, there is no consolidated overview of feature extraction techniques tailored to RNA sequences and structures. Existing approaches are scattered across domains, vary in implementation, and lack standardized documentation, hindering reproducibility and accessibility, particularly for researchers with limited programming expertise.

**Figure 1:**
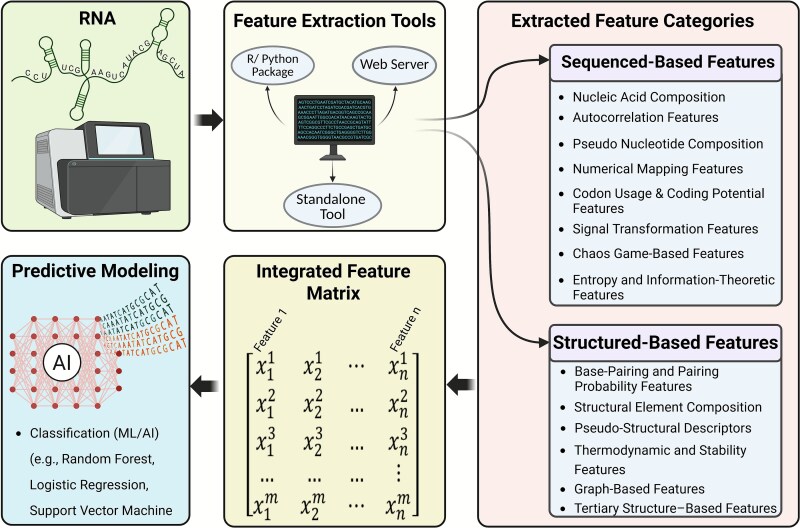
Overview of RNA feature extraction and machine learning workflow. RNA sequences are processed using standalone tools, web servers, or programming packages to extract informative numerical features. These features are categorized as sequence-based or structure-based, assembled into a feature matrix, and used to train machine learning models for various RNA-related predictive tasks.

To address this gap, this review provides a structured, accessible overview of established feature extraction strategies for RNA, categorized into sequence-based and structure-based methods. Figure 1 outlines the complete workflow, from raw RNA sequences through feature extraction and integration into predictive modelling frameworks. In addition to methodological categorization, we compile publicly available tools and software packages to support practical implementation. By organizing and contextualizing existing methods, this work aims to advance reproducibility, accessibility, and interpretability in ML-driven RNA biology.

## Traditional feature extraction from RNA

### Sequence-based features

Feature extraction from RNA sequences is a critical step in machine learning based RNA analysis, transforming raw nucleotide strings into structured quantitative representations suitable for predictive modelling. A wide range of feature extraction techniques have been developed to encode RNA sequences, spanning from simple frequency-based representations to advanced network-theoretic approaches [19], many of which are implemented in open-source toolkits [20]. Broadly, sequence-derived features can be categorised into the following groups: nucleotide composition-based features, numerical mapping and signal transformation methods, Fourier and Chaos-based features, entropy and information-theoretic measures, autocorrelation-based descriptors, pseudo nucleotide compositions, and similarity or instance-based features [19]. This categorization reflects an increasing level of computational and biological sophistication, progressing from the capture of local nucleotide patterns to the modelling of long-range dependencies, physicochemical properties, and structural complexities embedded within RNA sequences. A detailed summary of representative feature types, along with their computational demands, data requirements, example use cases, and limitations, is presented in Table 1.

**Table 1:** Comparative summary of sequence-based features for RNA machine learning applications.

Feature type	Description	Computational complexity	Data requirements	Example applications in RNA research	Limitations/notes
One-hot Encoding	Binary representation of nucleotides preserving sequence length.	$O(L)$ (Linear)	Raw sequence	Baseline for many models and used for classifying different types of RNAs, such as lncRNAs, circRNAs, and mRNAs.	Lacks biological context; scales poorly for long sequences
K-mer Frequency	Counts sub-sequences of length *k* to capture local patterns.	$O\left(L\times K\right)$ (Linear for constant k; polynomial if k varies with L)	Raw sequence	Applied in RNA type prediction (e.g. lncRNA, circRNA detection), subcellular localization, and ncRNA classification	Loses global sequence order; sensitive to *k* selection
Reverse Complement K-mer	K-mer frequency considering both original and reverse complement strand	$O\left(L\times K\right)$ (Linear for constant k; polynomial if k varies with L)	Raw sequence	RNA–RNA interaction studies	Slight information loss for self-complementary *k*-mers.
Mismatch Profile	Allows mismatches within *k*-mers (up to *m*).	$O\left(L\times{K}^m\right)$ (Polynomial for fixed mismatch parameter m; becomes exponential if m increases with k)	Raw sequence	Functional site and motif prediction, and RNA type classification	Larger feature space; needs dimensionality reduction.
Gapped K-mer (xxKGAP)	Uses sliding windows to count discontinuous nucleotide patterns with variable gaps	$O\left(L\times g\_\mathit{\max}\times K\right)$ (Linear in L when k and g_max are fixed; runtime increases proportionally with the number of gap values)	Raw sequence	Captures non-adjacent dependencies reflecting structural/functional RNA patterns	Exponential growth with k-mer size; complexity increases with gap size
GC Content	Proportion of guanine and cytosine nucleotides in sequence.	$O(L)$ (Linear)	Raw sequence	Differentiating coding versus non-coding regions	Simple metric; may not capture complex sequence patterns
AU/GC Ratio	Relative abundance of AU bases compared to GC bases.	$O(L)$ (Linear)	Raw sequence	Compositional analysis and sequence characterization	Single scalar value; limited discriminative power
GC Skew	Strand-specific nucleotide asymmetry measure: (G-C)/(G + C).	$O(L)$ (Linear)	Raw sequence	Originally for replication analysis; adapted for RNA functional classification	Developed for genomic analysis; limited RNA-specific validation
Enhanced Nucleic Acid Composition (ENAC)	Sliding-window local base composition.	$O\left(L\times w\right)$ (Linear when window size *w* is fixed; grows proportionally with *w* if *w* increases)	Raw sequence	Local compositional bias detection.	Requires fixed-length sequences.
ANF (Accumulated Nucleotide Frequency)	Encodes positional nucleotide occurrence profiles and density distribution.	$O(L)$ (Linear)	Raw sequence	Effective in studies on expression level prediction and splicing site detection	Less informative for distal dependencies
Z-Curve	Three-dimensional representation based on purine/pyrimidine, amino/keto, and hydrogen bond distributions.	$O(L)$ (Linear)	Raw sequence	Genome analysis adapted for RNA; captures multiple physicochemical properties	Originally designed for DNA; generates only 3 features
Numerical Mappings (Real/Integer/Complex)	Converts nucleotides to numerical values using different number systems.	$O(L)$ (Linear)	Raw sequence	Signal processing approaches and mathematical analysis	Arbitrary mapping; lacks biological interpretation.
EIIP Profiles	Encodes electronic properties relevant to bio-signalling.	$O(L)$ (Linear)	Raw sequence	Used in RNA–protein interaction studies and coding potential estimation	Less interpretable; dependent on biological assumptions
Autocorrelation Descriptors	Quantifies correlations in encoded properties at varying lags.	$O (L\times$ λ) (Linear when λ is fixed; grows proportionally with λ)	Encoded features (numeric property vectors)	Captures long-range dependencies; used in gene function annotation, subcellular localization prediction, and RNA type classification	May introduce redundancy; requires careful parameter tuning (lag size, property choice)
Pseudo K-tuple Composition (PseKNC)	Integrates sequence order with k-tuple frequencies.	$O (L\times$ *λ)* (Linear when λ is fixed; grows proportionally with λ)	Raw sequence + physicochemical property indices + tuning parameters (λ, ω)	Widely used in lncRNA subclassification, circRNA prediction, RNA editing site identification	Complex configuration: careful tuning required
Fickett Score	Integrates nucleotide composition with codon usage bias for coding potential	$O(L)$ (Linear)	Raw sequence	Distinguishing coding from non-coding RNAs	Requires codon-based analysis; limited to coding potential
Relative Codon Bias (RCB)	Quantifies non-uniform codon usage within open reading frames.	$O(L)$ (Linear)	Raw sequence	Codon usage analysis and coding sequence characterization	Applicable only to coding regions; requires ORF identification
ORF-Related Features	Max/average ORF length and coverage across multiple reading frames.	$O(L)$ (Linear)	Raw sequence	Distinguishing protein-coding from long non-coding RNAs	Limited effectiveness for lncRNA subtype classification
Fourier/CGR-Based Features	Transforms sequences into frequency or fractal-based visual domains.	$O\left(L\ \log L\right)$ (Quasi-linear)	Raw sequence	Used in sequence complexity analysis, regulatory RNA discovery, structural pattern exploration, and as inputs for deep learning pipelines	Poor interpretability; needs complementary features
Entropy-Based Features	Measures sequence randomness or uniformity.	$O(L)$ (Linear)	Raw sequence	Sequence complexity assessment; highlights conserved versus variable regions	Ignores structural or contextual sequence patterns

^*a*^ *Computational complexity expressed in Big-O notation describing runtime growth with sequence length (L) and method-specific parameters. **Parameters:** L = sequence length, k = k-mer size, m = mismatches allowed, g_max = maximum gap distance, λ = correlation lag, w = window size.*

#### Nucleic acid composition

These methods capture short range or local sequence order by counting the occurrence frequencies of adjacent or noncontiguous residues include:

##### One-hot encoding

One hot encoding is a widely adopted feature extraction technique that represents each nucleotide in the RNA sequence as a unique binary vector. An RNA sequence composed of the four bases A, U, C, and G can be represented by a 4-dimensional vector for each base. For example, A is represented as [1,0,0,0], U as [0,1,0,0], C as [0,0,1,0], and G as [0,0,0,1]. Therefore, an RNA sequence of length L can be expressed as a 4 × L dimensional binary matrix in which each column corresponds to a sequence position and each row represents a specific nucleotide [21].

##### K-mer composition

The k-mer feature counts the frequency of distinct nucleotide subsequences of length k within the RNA sequence. This is achieved by sliding a window of length k along the sequence and counting how often each possible k-mer appears. The process considers all contiguous subsequences of size k from position 1 to position (L − k + 1). The frequency (${\boldsymbol{f}}_{\boldsymbol{cs}}$) is calculated as:


(1) \begin{equation*} {f}_{cs}=\frac{\left(\frac{C_k}{4^{K-k}}\right)}{L-k+1} \end{equation*}

Where ${C}_k$ is the count of a specific k-mer, $L$ is sequence length, $K$ is the maximum $k$ value, and $4$ denotes the four nucleotide types [22]. K-mer features have been widely applied in the analysis of RNA sequence properties, including classification of coding and noncoding RNAs, identification of structural motifs, and functional annotation tasks [23–25].

##### Enhanced nucleic acid composition

Local nucleic acid composition can be calculated using the Enhanced Nucleic Acid Composition encoding, which applies a fixed length sliding window that moves sequentially from the 5′ to the 3′ end of the nucleotide sequence. This method is generally applied to nucleotide sequences of equal length. The sliding window size and sequence length determine the enhanced nucleic acid composition (ENAC) encoding dimension, calculated as $\left(\boldsymbol{sequence}\ \boldsymbol{length}-\boldsymbol{window}\ \boldsymbol{size}+\mathbf{1}\right)\times \mathbf{4}$. The ENAC encoding is defined as follows [26]:


(2) \begin{equation*} E=\left({b}_1,{b}_2,\dots, {b}_n\right), \end{equation*}

(3) \begin{equation*} b(i)=\frac{N_{(i)}}{N} \end{equation*}

$$ i\in \left\{A,C,G,T/U\right\} $$

Where $b(i)$ represents the frequency of nucleotide $i$ within a given window ($N$) of the sequence and ${N}_{(i)}$ is the count of nucleotide $i$ within that window.

##### Reverse complement k-mer

The reverse complement k-mer (k-RevcKmer) is a variation of the standard k-mer feature used in RNA sequence analysis. In this approach, both the original k-mers in the sequence and their reverse complements are considered during feature extraction. First, all possible k-mers are generated from the RNA sequence. Any k-mer i.e. identical to its reverse complement is removed to avoid redundancy. The remaining k-mers are then used to construct a feature vector, with each feature representing the frequency of a specific k-mer in the sequence. This method reduces the dimensionality of the k-mer space while retaining information from complementary strand orientations [27].

##### Mismatch profile

The mismatch profile approach is an extension of traditional k-mer counting that allows up to *m* mismatches within each k-mer, where *m* < *k*. For example, if *m* = 1 and *k* = 3, the notation [1, 3] refers to a 3-length subsequence with at most one mismatch. Considering a 3-mer ‘AAC’ with one allowed mismatch, the count would include not only ‘AAC’ itself, but also variants such as ‘AAG,’ ‘AAA,’ ‘AAU,’ ‘GAC,’ ‘CAC,’ and ‘UAC’ that appear in the sequence. The mismatch profile of a sequence *x* can be expressed as:


(4) \begin{equation*} {f}_{k,m}^{mis}(x)=\left(\sum_{j=0}^m{C}_{1,j},\sum_{j=0}^m{C}_{2,j},\dots, \sum_{j=0}^m{C}_{4^k,j}\right) \end{equation*}

Here, ${C}_{ij}$ indicates the frequency of the $i$-th k-mer variant in sequence $x$ with $j$ mismatches, where $i$ ranges from 1 to ${4}^k$ and $j$ from 0 to $m$. By incorporating both exact matches and near matches, the mismatch profile captures a broader spectrum of sequence patterns, potentially revealing biologically significant variations that standard k-mer counts may miss [28, 29].

##### x‌xKGAP encoding

The xxKGAP composition is a key approach employed in PyFeat package [7], considering kgaps in RNA sub-sequences. A sliding window is utilized to count the occurrences of discontinuous bases with g gaps (${\boldsymbol{C}}_{\boldsymbol{g}}$), and the frequency (${\boldsymbol{f}}_{\boldsymbol{ds}}$) is calculated as:


(5) \begin{equation*} {f}_{ds}=\left({C}_g/{4}^{G+2-g}\right)/\left(L-g-1\right) \end{equation*}

where $G$ represents the maximum value of $g$ [22]. For example, the sequence can be encoded into X_X frequencies for mMKGap features with a kgap of 1, producing 16-dimensional features ($4\times 1\times 4$). If kgap = 2, the sequence can be characterised by 32 features ($4\times 2\times 4$). For dMKGap, the total number of features is calculated as ${4}^2\times n\times 4$ [20]. This representation allows the capture of dependencies between nonadjacent nucleotides, which can reflect structural or functional patterns in RNA sequences.

##### Guanine–Cytosine (GC) content

GC content indicates the proportion of guanine and cytosine nucleotides within an RNA sequence. This metric is often employed to differentiate protein-coding regions from noncoding sequences. Generally, noncoding elements such as 5′ untranslated regions (UTRs) and introns have a lower percentage of GC bases compared to protein-coding sequences have a higher. The GC content is calculated as follows [30]:


(6) \begin{equation*} GC\ Content=\frac{N(G)+N(C)}{L_t} \end{equation*}

where $N(C)$ and $N(G)$ refer to the numbers of G and C nucleotides respectively, and ${L}_t$ is the overall transcript length.

##### Accumulated nucleotide frequency

The accumulated nucleotide frequency (ANF) encoding system represents the density and distribution of each nucleotide within a sequence [26]. To capture the nucleotide frequency and the distribution of each nucleotide in the RNA sequence, the density (${\boldsymbol{d}}_{\boldsymbol{i}}$) of any nucleotide (${\boldsymbol{S}}_{\boldsymbol{i}}$) at position $\boldsymbol{i}$ in the RNA sequence is defined using the following formula [31],


(7) \begin{equation*} {d}_i=\frac{1}{\left|{S}_i\right|}\sum_{j=1}^lf\left({s}_j\right), \end{equation*}

(8) \begin{equation*} f(q)=\left\{\begin{array}{@{}c}1\kern2.25em if\ {s}_j=q\\{}0\kern0.75em other\ cases\end{array}\right. \end{equation*}

Here, $l$ represents the length of the sequence, $\left|{S}_i\right|$ denotes the length of the $i$ -th prefix string $\left\{{s}_1,{s}_2,\dots{s}_i\right\}$ within the sequence, and $q\in \left\{A,C,G\ or\ U\right\}$. For the example sequence ‘UCGUUCAUGG’, the density of each nucleotide is as follows: For ‘U’, the density is 1 (1/1) at position 1, 0.5 (2/4) at position 4, 0.6 (3/5) at position 5, and 0.5 (4/8) at position 8. For ‘C’, the density is 0.5 (1/2) and 0.33 (2/6) at positions 2 and 6, respectively. The density of ‘A’ is 0.14 (1/7) at position 7. Finally, the density of ‘G’ is 0.33 (1/3) at position 3, 0.22 (2/9) at position 9, and 0.3 (3/10) at position 10 [31].

##### Adenine–Uracil to Guanine–Cytosine (AU/GC) ratio

The AU/GC ratio is a simple compositional feature that generates a single scalar value for each RNA sequence. It measures the relative abundance of adenine and uracil bases compared to guanine and cytosine bases. The ratio is calculated as [11].


(9) \begin{equation*} {{AU}}{/} _{{GC}}\ Ratio=\frac{\sum A+\sum U}{\sum G+\sum C} \end{equation*}

##### GC skew

GC skew, calculated as $ (G-C/\left(G+C\right)$, measures strand-specific nucleotide asymmetry and is commonly used to determine replication origin and terminus in bacterial genomes [27, 32]. Although originally developed as a genome level measure, GC skew can also be applied to RNA sequences to provide additional compositional information that may be relevant for distinguishing functional classes or structural properties [11, 33].

#### Autocorrelation descriptors

These approaches look for correlations between two di- or trinucleotides based on their physicochemical properties for RNA sequence analysis. Unlike simple compositional features, which only quantify nucleotide frequencies, autocorrelation descriptors preserve sequence-order information and can reveal periodic or long-range dependencies, making them useful for complex sequence analysis tasks. Two widely used approaches are autocovariance, which measures correlations of the same physicochemical property across nucleotide groups at a defined distance, and cross-covariance, which assesses correlations between different physicochemical indices [34]. According to the approaches applied in several studies for RNA, the autocorrelation module is divided into several categories based on different properties and correlation types. These include dinucleotide-based autocorrelation (DAC), dinucleotide-based Moran autocorrelation (DMAC), dinucleotide-based Geary autocorrelation (DGAC), and normalised Moreau-Broto autocorrelation (NMBAC). Similarly, for cross-correlation and auto-cross-correlation modules, two methods exist for RNA: dinucleotide-based cross-correlation (DCC) and dinucleotide-based auto-cross-correlation (DACC) [35, 36].

#### Pseudo nucleotide composition

The third category of sequence-derived features includes pseudo k-tuple nucleotide composition (PseKNC) methods, which are designed to capture both global and long-range sequence-order information, as well as physicochemical properties of nucleotides. Due to their strong performance across various predictive tasks, several versatile web servers and software tools have been developed to generate pseudo nucleotide composition features [37–39]. A comprehensive overview of pseudo nucleotide composition approaches can be found in a recent review [40]. Within this category, pseudo dinucleotide composition (PseDNC) encoding is one of the most widely used methods in RNA sequence analysis. PseDNC takes into account not only the sequential arrangement of nucleotides but also the physicochemical properties of dinucleotide pairs within the RNA molecule, resulting in a numerical feature set for each analyzed sequence. The total number of PseDNC features is given by 16 + λ. The initial 16 features are derived from pairs of adjacent dinucleotides. The remaining λ features are calculated based on dinucleotide pairs that are separated by different distances along the sequence. λ denotes the greatest possible separation between any two dinucleotides considered in the analysis [41]. Several publicly available packages have been developed to extract PseDNC features such as Pse-in-One 2.0 [42], repRNA [43], and UltraPse [44].

#### Numerical mapping features

##### Real, integer, and complex number mappings

In sequence analysis, numerical mapping methods such as integer, complex, and real number representations are widely used to convert symbolic nucleotide sequences into numerical form suitable for computational analysis [19]. Integer mapping assigns simple whole numbers to nucleotides, e.g. A = 0, C = 1, G = 2, and T/U = 3 [45]. Complex number mapping places nucleotides as points in the complex plane, such as A = 1 + i, T/U = 1 − i, C = −1 − i, and G = −1 + i [46]. Real number mapping, on the other hand, uses continuous real values such as A = −1.5, T/U = 1.5, C = 0.5, and G = −0.5. This representation has the useful property that complementary sequences can be derived by reversing the sequence order and changing the sign of each value [47].

##### Electron-ion interaction pseudopotentials

EIIP encoding transforms RNA sequences into numerical feature vectors by assigning each nucleotide a specific electron-ion interaction pseudopotentials (EIIP) value: A = 0.1260, C = 0.1340, G = 0.0806, and U = 0.1335 [48]. To represent trinucleotide composition, this method constructs a 64-dimensional feature vector in which each element corresponds to a specific trinucleotide. For a trinucleotide sequence $\boldsymbol{mno}$, the EIIP value is calculated as:


(10) \begin{equation*} {\boldsymbol{EIIP}}_{\boldsymbol{m}\boldsymbol{no}}={\boldsymbol{EIIP}}_{\boldsymbol{m}}+{\boldsymbol{EIIP}}_{\boldsymbol{n}}+{\boldsymbol{EIIP}}_{\boldsymbol{o}} \end{equation*}

where m, n, o ∈ {A, C, G, U} and ${f}_{mno}$ is the frequency of that trinucleotide in the sequence. The resulting vector is:


(11) \begin{align*} D=\left[{EIIP}_{AAA}\times{f}_{AAA},{EIIP}_{AAC}\times{f}_{AAC},{EIIP}_{AAA}\right.\notag\\\left.\times{f}_{AAA},\dots ..,{EIIP}_{UUU}\times{f}_{UUU}\right] \end{align*}

##### Z-curve

The Z-curve theory, originally developed for DNA sequence analysis, is a three-dimensional representation of a sequence’s base distribution [49]. This method can be effectively adapted for RNA sequence analysis due to its distinct geometrical properties and the similarity between RNA and DNA nucleotide structures, with the primary difference being the substitution of uracil (U) for thymine (T) [11, 50, 51]. The Z curve is formed by a series of nodes, P_0_, P_1_, …P_N_, where N is the sequence length and each node has coordinates ${\boldsymbol{X}}_{\boldsymbol{n}}$, ${\boldsymbol{Y}}_{\boldsymbol{n}}$, ${\boldsymbol{Z}}_{\boldsymbol{n}}$ defined as:


(12) \begin{equation*} {x}_n=\left({A}_n+{G}_n\right)-\left({C}_n+{U}_n\right) \end{equation*}

(13) \begin{equation*} {y}_n=\left({A}_n+{C}_n\right)-\left({G}_n+{U}_n\right) \end{equation*}

(14) \begin{equation*} {z}_n=\left({A}_n+{U}_n\right)-\left({C}_n+{G}_n\right) \end{equation*}

$$ n=0,1,2,\dots, N $$

Where ${A}_n$, ${G}_n$, ${C}_n$, ${U}_n$ denote the cumulative counts of each nucleotide from the first position up to position *n* in the sequence.

Nucleotides are classified into six categories based on their properties: purine (R = A, G) versus pyrimidine (Y = C, U), amino (M = A, C) versus keto (K = G, U), and hydrogen bond strength, strong (S = G, C) versus weak (W = A, U). The x-component of the Z-curve represents the distribution of purines and pyrimidines, the y-component corresponds to amino and keto distribution, and the z-component reflects the distribution of strong and weak hydrogen bonds in the nucleotide sequence [52]. As a result, three numerical features can be generated from the Z-curve representation for downstream analysis.

#### Codon usage and coding potential features

##### Fickett score

The Fickett score is a feature extraction method designed to differentiate coding from noncoding RNAs by integrating nucleotide composition with codon usage bias. It evaluates four position values and four content values for each sequence followed by a weighted summation. The position values capture the preference of each nucleotide (A, C, G, U) for specific positions within codons, offering insights into positional biases within the transcript. For each nucleotide, its position value within the RNA transcript is determined using the following formula:


(15) \begin{equation*} {A}_1=N\left( base\ A\ in\ Position\ 0,3,6,\dots \right) \end{equation*}

(16) \begin{equation*} {A}_2=N\left( base\ A\ in\ Position\ 1,4,7,\dots \right) \end{equation*}

(17) \begin{equation*} {A}_3=N\left( base\ A\ in\ Position\ 2,5,8,\dots \right) \end{equation*}

(18) \begin{equation*} {A}_{pos}=\frac{\mathit{\operatorname{Max}}\ \left({A}_1,\kern0.5em {A}_2,\kern0.5em {A}_3\right)}{\mathit{\operatorname{Min}}\ \left({A}_1,\kern0.5em {A}_2,\kern0.5em {A}_3\right)+1} \end{equation*}

Here, $N\left(\right)$ represents the total count of nucleotides under the specified condition. The values for ${U}_{pos}$, ${G}_{pos}$, ${C}_{pos}$ are derived in the same way as ${A}_{pos}$. The overall content-based metrics for each nucleotide in the transcript are then computed as follows:


(19) \begin{equation*} {A}_{content}=\frac{N\left( base\ A\ in\ an\ RNA\ transcript\right)}{L_t} \end{equation*}

The calculation methods for ${U}_{content}$, ${G}_{content}$, and ${C}_{content}$are identical. Ultimately, a lookup table is employed to transform the four positional attributes and four compositional attributes into probabilities indicative of coding potential. The Fickett score is then derived by multiplying these eight probability values (p) by their respective weighting factors (w). These weights reflect the effectiveness of each positional or compositional feature in distinguishing between coding and noncoding sequences [30, 53].


(20) \begin{equation*} Fickett\ score=\sum_{i=1}^8{p}_i{w}_i \end{equation*}

##### Relative codon bias

Relative codon bias (RCB) serves as a metric to quantify the non uniform usage of codon triplets within the open reading frames (ORFs) of an RNA transcript. It measures how much the observed codon usage deviates from what would be expected based on the independent nucleotide composition at each codon position. To derive the RCB value for an ORF, the product of the individual usage biases for all its codon triplets is computed. The codon usage bias ${\boldsymbol{d}}_{\boldsymbol{xyz}}$ for a specific triplet $\left(\boldsymbol{x},\boldsymbol{y},\boldsymbol{z}\right)$ is determined as follows:


(21) \begin{equation*} {d}_{xyz}=\frac{f\left(x,y,z\right)-{f}_1(x){f}_2(y){f}_3(z)}{f_1(x){f}_2(y){f}_3(z)} \end{equation*}

(22) \begin{equation*} f\left(x,y,z\right)=\frac{N\left(x,y,z\right)}{L_{codon}} \end{equation*}

(23) \begin{equation*} {f}_1(x)=\frac{N\left( base\ x\ in\ position\ 0\ of\ each\ codon\right)}{L_{codon}} \end{equation*}

Here, $N\left(x,y,z\right)$ is the count of the codon triplet $\left(x,y,z\right)$ found in the ORF, and ${L}_{codon}$ is the ORF length in codons. The calculations for ${f}_2(y)$ and ${f}_3(z)$ at the second and third positions are analogous to the calculation of ${f}_1(x)$ at the first nucleotide position. Subsequently, the complete RCB value for the RNA transcript’s ORF is computed as shown below [30, 54]:


(24) \begin{equation*} RCB={\left(\prod_{i=1}^{L_{codon}}\left(1+{d}_{xyz}^i\right)\right)}^{1/L}-1 \end{equation*}

##### ORF related features (max ORF length, max ORF coverage, average ORF length, average ORF coverage)

An open reading frame (ORF) is a segment within an RNA transcript that has the potential to encode a protein. Analyses of ORF characteristics are commonly used to distinguish protein-coding transcripts from long noncoding RNAs (lncRNAs), although these features are generally less effective for differentiating among various lncRNA subtypes [10]. To capture protein-coding information more comprehensively, analyses may extend beyond the conventional definition of an ORF as the region between a start codon and a stop codon. Alternative definitions include ORFs that begin with a start codon and extend to the transcript’s end, or segments that span from any nonstop codon to a stop codon. An integrated approach can also select the longer sequence between these start-codon-focused and stop-codon-focused variants. For each ORF type, the maximum length across all three reading frames can be determined and extracted as the max ORF.

In addition to their absolute length, the max ORF coverage can be computed by dividing the max ORF length by the total transcript length. Furthermore, for conventionally defined ORFs (bounded by start and stop codons), both the average ORF length and the average ORF coverage are determined. These features provide informative measures of coding potential and have been widely applied in computational transcript classification [30].

#### Signal transformation features (Fourier-based)

This category encompasses feature extraction techniques that convert RNA sequences into numerical signals and then apply methods from genomic signal processing (GSP) to derive informative features. Among these, the Fourier Transform (FT) is one of the most widely used approaches in biological sequence analysis [10, 55, 56]. A detailed mathematical formulation of the Fourier-based approach for nucleotide sequences is provided in [10].

#### Chaos game-based features

Chaos game representation (CGR) is a method that visually encodes RNA sequences as two-dimensional fractal patterns derived from nucleotide composition. For machine learning applications, CGR can be quantified using frequency chaos game representation (FCGR), in which the fractal image is divided into a grid and the frequencies of subsequences falling into each grid cell are counted. This process generates a numerical matrix that can be flattened into a fixed-length vector, providing an alignment-free feature representation for RNA sequence analysis. The detailed methodology and applications of CGR and FCGR are described in [57].

#### Entropy and information-theoretic features

Several studies have applied concepts from information theory to extract meaningful features from biological sequences, with Shannon entropy (SE) being one of the most widely used measures [58, 59]. SE quantifies the uncertainty or diversity in the distribution of nucleotides or k-mers within a sequence, providing insights into its complexity. In addition to SE, Tsallis entropy (TE) [60, 61] has been successfully employed as an alternative or complementary descriptor in sequence analysis. TE generalizes the concept of entropy by introducing a parameter that can adjust the sensitivity of the measure to rare or frequent events. Both SE and TE capture important statistical properties of RNA sequences and can be applied at different k-mer levels to highlight sequence variability and compositional bias [30].

### Structural feature extraction

Understanding the structural configuration of an RNA molecule is an essential first stage in uncovering its functional mechanisms [62]. Among structural characteristics, the secondary structure is particularly critical in diverse biological processes and is often more conserved than the primary sequence [63]. The set of base pairs formed through hydrogen bonding between nucleotides defines the RNA secondary structure. The main challenge in secondary structure prediction lies in determining which nucleotides are paired with each other in a given sequence [64]. Thermodynamic principles can be used to predict the secondary structure of an RNA sequence [65]. These thermodynamics-based methods employ nearest-neighbour parameters to estimate structural stability, which is quantified by the change in folding free energy [66–68]. Structure prediction is commonly achieved by determining the conformation with the lowest free energy [62]. Minimum free energy (MFE) acts as a fundamental structural indicator, reflecting the stability of the RNA structure [63]. The assumption is that a lower free energy implies greater stability of the RNA secondary structure [30]. Alternative prediction strategies include sampling from the Boltzmann ensemble to identify a representative centroid structure [69] or selecting the structure with the highest sum of base-pairing probabilities, known as the maximum expected accuracy (MEA) structure [70].

A widely used tool for RNA secondary structure prediction is RNAfold, part of the ViennaRNA Package, which applies MFE calculations to identify the most probable configurations [71]. RNAfold decomposes RNA secondary structures into elements such as interior loops, hairpin loops, multiloops, bulge loops, and stacking pairs, with each contributing to the total free energy. The total free energy of RNA’s secondary structure is determined by summing the free energy values of its constituent substructures. The most stable predicted structure is generated for each RNA transcript and used for downstream feature extraction [71]. A comprehensive list of available RNA secondary structure prediction tools is available at [72].

In a study conducted by Kang et al. [22], RNA secondary structures were predicted using the RNAfold package, which represents structural features using a system of brackets (‘( “or’ )” = paired nucleotide) and dots (‘.’ = unpaired). These approaches can extract both continuous and discontinuous structural patterns. However, unlike sequence-based analyses that consider the four nucleotide types, structural analysis is constrained to two symbol types (brackets and dots), necessitating adjustments in calculation parameters. Accordingly, several structural features can be derived from the dot-bracket notations produced by these tools, as described in the following paragraphs.

A comprehensive summary of these structural feature types, their computational profiles, and their relevance across RNA research domains is provided in Table 2.

**Table 2:** Comparative summary of structure-based features for RNA machine learning applications.

Feature type	Description	Computational complexity	Data requirements	Example applications in RNA research	Limitations/notes
Minimum Free Energy (MFE)	Represents the thermodynamic stability of the most likely predicted 2D structure.	$O\left({L}^3\right)$ (Cubic)	RNA sequence	Predicting RNA stability, siRNA efficacy, antisense design, validating structural motifs	Assumes lowest energy = most likely conformation; may not reflect dynamic or *in vivo* state
Numerical Descriptors (from 2D structure)	Counts and ratios of structural elements (e.g. paired ratio, loop counts, GC content in stems).	$O(L)$ (Linear)	Dot-bracket structures	RNA class differentiation (e.g. miRNA versus tRNA), functional fold prediction, benchmarking	Heavily depends on accurate structure prediction; may oversimplify complex topologies
Triplet Structure-Sequence	Integrates local sequence identity with structural context using 3-nucleotide windows.	$O(L)$ (Linear)	Sequence + dot-bracket structure	miRNA identification, high-performance classification tasks	Sensitive to structure prediction accuracy; limited to local patterns
Pseudo-Structure Status Composition (PseSSC/PseDPC)	Captures compositional and sequential information using correlation functions based on structure status.	$O(L\times$ λ) (Linear when λ is fixed; increases proportionally with λ)	Sequence + structure + correlation parameters	Stem-loop analysis, structural motif identification	Parameter λ selection critical; requires accurate secondary structure
Loop Structure Counts & Coverage	Number and coverage ratios of different loop types (hairpin, interior, bulge, multibranch).	$O(L)$ (Linear)	Dot-bracket structures	Structural classification, stability assessment	Simple metrics; may not capture structural complexity
Topological Descriptors (from graphs)	Uses graph-theoretic features from RNA structure graphs like centrality, clustering, graphlets.	$O\left({L}^2\right)$ (Quadratic) to $O\left({L}^3\right)$(Cubic)	Graph representation of RNA structure	Functional RNA classification, structure-based motif discovery, structure–function modeling	Computationally expensive; abstract features; interpretability challenges
Graph Kernels	Kernel functions measuring similarity between RNA structure graphs using random walks.	$O\left({L}^4\right)$ (Quartic)	Graph representations for all sequences	Family-level classification, structural similarity analysis, kernel-SVM applications	Scales poorly with dataset size; computationally intensive; kernel selection critical
Graph Embedding (for networks)	Learns low-dimensional embeddings from RNA molecules in biological interaction networks.	$O\left(E\times d\times iter\right)$ (Linear in E when d and iter are fixed; grows proportionally with embedding size and training iterations)	RNA interaction networks (e.g. circRNA-miRNA)	RNA-miRNA and RNA-protein interaction prediction, network hub discovery	Performance depends on network quality; limited to known entities
Tertiary (3D) Structure Features	Captures atomic-level spatial and physicochemical descriptors from experimentally resolved structures.	$O\left({L}^3\right)$ (Cubic)	High-resolution 3D models (PDB files)	Binding site prediction, aptamer modeling, RNA-based drug discovery	Scarcity of experimental structures; computationally expensive
Data-Driven Structure Refinement	Incorporates experimental probing data (SHAPE, DMS) to refine structure predictions.	$O\left({L}^3+{N}_{exp}\right)$ (Cubic folding complexity plus a linear experimental-data term)	Experimental probing data + RNA sequence	Structure refinement, accuracy improvement, viral RNA annotation	Requires experimental data; quality varies; not standardized

^*a*^ *Computational complexity expressed in Big-O notation describing runtime growth with sequence length (L) and method-specific parameters. **Parameters:** L = sequence length, λ = correlation lag parameter, E = number of edges in the RNA interaction network, d = embedding dimensions, iter = algorithm iterations.*

#### Paired ratio

This is a metric based on the secondary structure of an RNA transcript, representing the proportion of nucleotides involved in Watson-Crick base pairing compared to those that remain unpaired. This ratio is used to assess structural stability; RNA molecules with a higher percentage of paired nucleotides have more stable secondary structures [30]. The formula is as follows:


(25) \begin{equation*} Paired\ Ratio=\frac{N\left( Paired\ Nucleutide\ Bases\right)}{L_t} \end{equation*}

#### Triplet

This method integrates both sequence and structure information and has shown superior performance in tasks such as microRNA identification [43, 73]. Using dot/bracket notation, there are eight [23] possible structural configurations for a set of three adjacent nucleotides: ‘(((‘, ‘((.’, ‘(..’, ‘(.(‘, ‘.((‘, ‘.(.’, ‘..(‘, and ‘ …’. By focusing on the middle nucleotide within each group of three, 32 possible structure-sequence combinations (4 × 8) can be obtained, denoted as f_A_ (‘(((‘), f_G_ (‘(((‘), etc. These combinations define the triplet structure-sequence elements, which integrate both nucleotide sequence and corresponding structural information, allowing for comprehensive analysis [21, 43].

#### Pseudo-structure status composition and pseudo-distance structure status pair composition

*Liu et al.* proposed pseudo-structure status composition (PseSSC) and pseudo-distance structure status pair composition (PseDPC) methods for capturing the compositional and sequential information of RNA sequences by efficiently representing RNA secondary structures like stem loops. These approaches approximate the sequential information of RNA sequences employing a correlation function based on secondary structure status, considering both the distance between structural status pairs and the minimum free energy [74]. Details can be found in the referenced sources [75, 76].

#### Number of distinct loop structures

**Figure 2:**
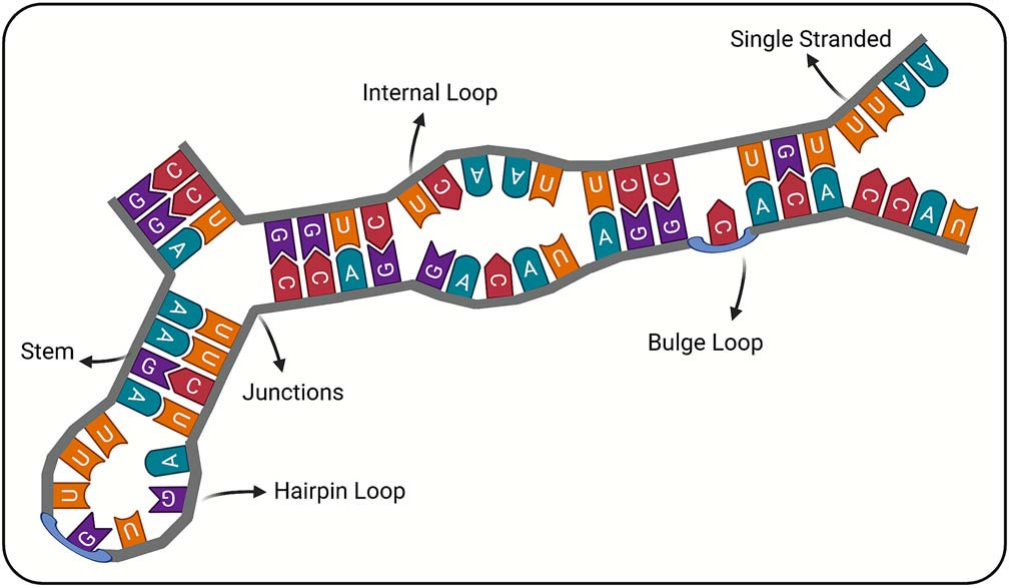
Example of an RNA secondary structure illustrating various types of structural elements. (created in BioRender.com).

This metric counts different loop types in the secondary structure, including interior loops (N(I)), hairpin loops (N(H)), bulge loops (N(B)), and multibranch loops (N(M)) [30]. The typical loop structures are illustrated in Fig. 2.

#### Coverage of different loop structures

For each loop type, coverage is computed as the number of loops divided by the transcript length [30].


(26) \begin{equation*} C(H)=\frac{N(H)}{L_t} \end{equation*}

(27) \begin{equation*} C(I)=\frac{N(I)}{L_t} \end{equation*}

(28) \begin{equation*} C(B)=\frac{N(B)}{L_t} \end{equation*}

(29) \begin{equation*} C(M)=\frac{N(M)}{L_t} \end{equation*}

#### GC content of paired nucleotides

This attribute is calculated as the proportion of guanine-cytosine (G-C) base pairs in the secondary structure of an RNA transcript. G-C bonds are stronger and more stable than adenine-thymine/uracil (A-T/U) bonds, so a higher GC content in paired nucleotides typically reflects a more stable secondary structure for the RNA transcript [30].


(30) \begin{equation*} GC\ content\ paired\ nucleotides=\frac{N\left( Paired\ G\right)+N\left( Paired\ C\right)}{N\ \left( paired\ nucleotides\right)} \end{equation*}

#### Advanced structural representations: graph-based features

Beyond deriving numerical counts from dot-bracket notation, a more powerful paradigm models RNA secondary structures and their interaction networks as graphs. This allows the extraction of sophisticated features that capture the topology and relational context of the RNA molecule.

##### Topological descriptors as engineered features

In this approach, an RNA secondary structure is represented as a graph where nucleotides are nodes, and both the phosphodiester backbone and base-pairing interactions form the edges [77]. From this representation, a vector of numerical features can be engineered using classical graph-theoretic metrics. These descriptors can include node centrality measures, clustering coefficients, and the frequency of small, recurring subgraphs known as graphlets [78]. These feature vectors, which describe the RNA’s topology, can then be used as direct input for standard classifiers.

##### Graph kernels for similarity-based classification

Graph kernels offer a way to compare RNA secondary structures without explicit feature extraction by computing similarity directly between graph representations [79].

One such approach is the marginalized graph kernel, which has been applied to RNA sequences represented as ‘labelled dual graphs’ [80]. In this representation, helical regions of the RNA structure are treated as nodes and the connecting loops as edges. The kernel then computes a similarity score between two RNA graphs by comparing the sets of all possible random walks (paths) within each graph. While expressive, it is computationally intensive and sensitive to structure prediction noise, limiting scalability [79].

##### Graph embedding for network-based features

When analyzing interaction networks (e.g. RNA–RNA or RNA-protein networks), graph embedding algorithms provide a way to encode the position and role of each RNA within the larger network. These methods learn a low-dimensional vector representation (an ‘embedding’) for each node (RNA molecule). An example is the HOPE (High-Order Proximity preserved Embedding) algorithm, which generates feature vectors that capture complex network relationships [81]. As demonstrated by Zhou et al. (2024) [82], these structural embeddings can serve as powerful features for traditional classifiers like Gradient Boosted Decision Trees (GBDT) to predict interactions, such as those between circRNAs and miRNAs. This approach effectively converts the global topology of a biomolecular network into a fixed-length feature vector for each RNA.

#### Integrating experimental data for data-driven structural feature extraction

One of the limitations of the methods described above is their reliance on purely *in silico*, thermodynamics-based predictions, which may not always reflect the true biological structure. To overcome this, ‘data-driven’ approaches incorporate direct experimental evidence to produce more biologically faithful structural models, from which higher-quality features can be extracted.

##### Refining secondary structures with chemical probing

This approach uses experimental data to guide 2D folding algorithms. High-throughput chemical probing techniques, such as SHAPE (Selective 2’-Hydroxyl Acylation Analyzed by Primer Extension) and DMS (dimethyl sulfate), provide per-nucleotide reactivity scores that indicate which bases are flexible and likely unpaired [83]. This information can be integrated as a pseudo-energy term to constrain folding algorithms, significantly improving prediction accuracy [84].

The GraphClust2 framework is a prime example of a tool that leverages this principle. It is designed to support structure-probing data from SHAPE and DMS experiments, generating ‘SP-directed structure graphs’ that lead to more accurate clustering and annotation of RNA families [85]. Similarly, the QRNAstruct method uses sequence-activity data (such as SHAPE reactivities) to learn which position-specific structural features are most relevant to a given biological function, demonstrating that experimentally informed features yield more ‘plausible trends in RNA secondary structure’ [83].

#### Deriving features from tertiary (3D) structures

Tertiary (3D) structure provides detailed representation of RNA architecture and is obtained through experimental techniques such as X-ray crystallography, NMR spectroscopy, and cryo-electron microscopy [86]. Databases like RNAsolo curate, clean, and standardize these experimentally resolved 3D structures from the Protein Data Bank, making them accessible for computational studies [87].

When 3D structural data are available, they allow the extraction of rich spatial features, such as solvent accessible surface area [86] and torsion angles along the sugar–phosphate backbone [88]. These features are indispensable for machine learning models aimed at predicting molecular interactions, such as RNA-protein or RNA-ligand binding sites, as these processes are governed by the 3D shape and chemistry of the RNA surface [89]. While the scarcity of high-resolution 3D RNA structures remains a limitation, the use of these features is a critical and growing area of RNA bioinformatics.

## Comparative impact of feature set choice on model performance

The predictive performance of machine learning models in RNA analysis is strongly influenced by the composition of the input feature set [76, 90]. While the choice of algorithm plays a role, the diversity and informativeness of the features are equally critical in determining model accuracy. To provide a detailed examination of this effect, we synthesize the evidence from four independent studies that employed different combinations of RNA-derived features for various classification tasks, with a full breakdown provided in Table 3.

**Table 3:** A detailed comparison of feature set performance synthesized from four independent studies

Study / reference	Prediction task / aim	Model used	Feature sets compared	Performance metric	Key results and comparison
A) Jha et al. (2022) [91]	Predicting bacterial (*Salmonella typhimurium* LT2 (SLT2) small RNAs (sRNAs)	AdaBoost	Individual Groups (G1–G15): G1: seven biologically derived features G2–G15: Sequence-derived features including k-mer, reverse complement k-mer (RCkmer), and PseKNC variants Combined Group (G1–15): Total of 2229 features comprising: 7 biological features 2222 sequence-derived features	AUROC	**Combined Feature Set (G1–15):** Achieved the highest performance with AUROC = 0.954 ± 0.02 **Top Individual Performers:** G1 (Biological features): AUROC = 0.938 ± 0.039 G11 (5-RCkmer): AUROC = 0.881 ± 0.043 **Lowest Performing Feature:** G7 (1-RCkmer): AUROC = 0.652 ± 0.07 **Key Insight:** Integrating diverse feature types provides a clear performance advantage.
B) Khalid et al. (2023) [92]	Predicting disease-related lncRNAs	SVM	Individual types: 2-mer, 3-mer, PseDNC, and structural features. Combined: A set of eight feature types including 2mers, 3mers, PseDNC, Conservation score, GC content, lncRNA protein-interaction features, mutation count, structural features	Accuracy	Fully integrated feature set significantly outperformed all individual sets with an accuracy of 76% Individual feature sets showed accuracy ranging from 64% to 71% **Key Insight:** Demonstrates a substantial performance gain achieved through strategic feature integration
C) Li et al. (2024) [93]	Predicting lncRNA subcellular localization (nucleolus versus nucleoplasm)	Random Forest	Compared 4 individual feature categories: Autocorrelation, Pseudo-nucleotide composition, Nucleic acid composition, and Binary encoding. Fused Feature Set Evaluated: Combination of top three single features: ENAC + Binary + ANF	AUROC	**Best Individual Features:** **ENAC** achieved the highest performance with **AUC** = 0.851 **Binary:** AUC = 0.838 **ANF:** AUC = 0.820 **Fused Feature Set:** **ENAC + Binary + ANF:** AUC = 0.827 **Key Insight:** A single, highly informative feature (ENAC) outperformed a simple fusion of multiple features. This suggests that naive feature concatenation may introduce redundancy, reducing model performance.
D) Tang et al. (2018) [94]	Predicting bacterial sRNAs	Random Forest	Compared 17 Individual Feature Groups (F1–F17): Spectrum Profile (k-mer): F1–F5 k = 1 to 5 Mismatch Profile: F6–F8 Varying mismatch settings Reverse Complement k-mer (RevcKmer): F9–F13 Captures strand-complementary patterns Pseudo Nucleotide Composition (PseKNC Variants): F14–F17 Includes features like PCPseDNC and others	AUROC	**Performance varied widely across individual feature types** **Top Performers:** 4-RevcKmer (F12): AUC = 0.938 5-RevcKmer (F13): AUC = 0.937 **Weakest Performer:** 1-RevcKmer (F9): AUC = 0.632 **General Trend:** Performance increased with greater k-mer complexity: 1-mer: AUC = 0.682 4-mer: AUC = 0.923

A general pattern that emerges is the superior performance of models trained on integrated feature sets that combine multiple descriptor types. This is clearly demonstrated in the prediction of bacterial sRNAs by Jha et al. (2022) [91]. Their analysis revealed that a combined set of biological and sequence-derived features achieved the highest AUROC of 0.954 ± 0.02. Interestingly, their study also showed that a set of just seven biologically-derived features (G1) performed nearly as well (AUROC = 0.938 ± 0.039), underscoring the high information content of domain-specific knowledge. This principle of beneficial integration is further supported by the work of Khalid et al. (2023) [92] on disease-related lncRNAs, where a fully integrated feature set achieved a final accuracy of 76%, a substantial improvement over the best-performing individual feature set (3-mers at 71%).

However, a more detailed analysis reveals that naive feature fusion is not universally optimal. The study by Li et al. (2024) [93] on lncRNA localization provides a compelling counter-example. Here, the single best-performing feature, ENAC (Enhanced Nucleic Acid Composition) (AUC = 0.851), outperformed a direct concatenation of the top three individual features, which achieved a lower AUC of 0.827. This result suggests that without careful feature selection, simply combining features can introduce redundancy that may degrade model performance.

Furthermore, the choice of individual features is itself a critical factor, as their predictive power can vary dramatically. The work by Tang et al. (2018) [94] highlights this wide performance variance among 17 different sequence-derived feature types. In their study, more complex descriptors like 4-RevcKmer (AUC = 0.938) demonstrated high predictive power, whereas simpler ones like 1-RevcKmer (AUC = 0.632) were far less effective. This emphasizes the importance of selecting potent individual features before considering their combination.

Together these studies highlight an important insight that integrating complementary feature types can enhance model performance, although the best approach depends heavily on the specific context. Simply combining all available descriptors does not guarantee optimal results. Instead, the most effective feature set is one i.e. carefully designed and selectively chosen based on the biological question at hand. This underscores the critical role of thoughtful feature engineering as a foundational step in developing robust and reliable predictive models in RNA biology.

## Feature extraction tools

Over the years, a variety of computational tools have been developed to facilitate the extraction of RNA sequence and structure features. These tools implement a broad spectrum of methodologies, enabling the derivation of descriptors such as k-mer frequencies, physicochemical properties, structural stability metrics, entropy-based measures, and other specialized attributes discussed in previous sections. Table 4 presents a summary of widely used tools in the literature, outlining their primary functionalities and feature categories. The availability of these resources has significantly streamlined the process of generating high-dimensional, informative feature sets for downstream machine learning applications in RNA biology.

**Table 4:** A comparative analysis of publicly available tools for RNA feature extraction in machine learning applications

Tool name/ year	Platform	Feature types supported	RNA modalities supported	Scalability	Ease of use (if accessible)	Compatibility	Maintenance status	Strengths / when to prefer	Limitations
repRNA 2016 [43]	Web Server	k-mer, pseudo-nucleotide, structure-based features	All RNA types	**Low** (50-sequence limit)	**High** No-code web interface	**Standard Input:** Accepts standard FASTA format.	Likely Unmaintained (URL often defunct)	Good for non-programmers needing to generate PseKNC or pseudo-structural features for a small, curated dataset.	**Scalability:** Unsuitable for any large-scale analysis. **Automation:** Lacks programmatic access for pipeline integration. **Availability:** Unreliable/likely offline.
PseKNC 2014 [37]	Web Server	Pseudo K-tuple nucleotide composition with physicochemical properties	All RNA types	**Low** (100-sequence limit)	**High** No-code web interface/ Extensive parameter control	**Standard Input:** Accepts standard FASTA format.	Offline / Deprecated	Customizable PseKNC feature generation	No longer accessible/ PseKNC-only features/ Not scalable for large datasets
PseKNC-General 2015 [38]	Standalone (GUI & Command-line)	K-tuple nucleotide composition; autocorrelation descriptors; pseudo-nucleotide composition	All RNA types	**High** (No data-size limit)	**High** GUI for non-programmers/ Command-line for scripting	**Standard** **Input:** Accepts standard FASTA format.	Offline / Deprecated	Rich feature set/ user-defined indices	Official download link is defunct/ Not a native R/Python library
BioTriangle 2016 [95]	Web Server	Nucleic acid composition, autocorrelation, pseudo-features, interaction descriptors	All RNA types	**Low to Moderate** As a web server, it is inherently limited by server-side processing capacity	**High** No-code, modular interface	**Standard** **Input:** Accepts standard FASTA format	Functional	Ideal tool for generating combined feature vectors for pairs of interacting molecules (e.g. RNA-protein, RNA-ligand)/ no coding required	Lacks a command-line version, preventing integration into scalable, automated pipelines/ No Structure-Based RNA Features
BioSeq-Analysis2.0 2019 [96]	Web Server & Standalone Tool	90+ sequence-level & 26 residue-level descriptors; auto ML integration	All RNA types	**High** (Standalone version*)	Web & CLI; end-to-end ML support	**Standard** **Input:** Accepts standard FASTA format	Functional	Automated pipelines; great for both beginners and experts	Default settings may limit flexibility in some tasks
BioSeq-Analysis 2019 [34]	Web Server & Standalone Tool	Nucleic acid composition; autocorrelation descriptors; pseudo-nucleotide composition; predicted structure composition	All RNA types	**High** (Standalone version)	**High** Automated predictor-building pipeline/ Dual GUI & CLI access	**Standard** **Input:** Accepts standard FASTA format	Superseded by 2.0 Version (Offline)	Automated Model Building	Entirely replaced by the more advanced BioSeq-Analysis2.0
Nfeature 2021 [36]	Web Server & Standalone Tool	14 k + features (composition, autocorrelation, entropy, repeat indices)	All RNA types	**High** (Standalone version)	**High** No-code web interface/ Standalone for pipeline integration	**Standard** **Input:** Accepts standard FASTA format	Functional	Huge feature diversity; ideal for exploratory feature mining	Requires feature reduction; sequence-based only
iLearn 2020 [97]	Web Server & Python Toolkit	Nucleic acid composition; binary encoding; position-specific trinucleotide tendencies; autocorrelation; pseudo-composition	All RNA types	**High** (Python toolkit	**High** Web server for accessibility/ Python-native for script integration/ Automated ‘best model’ selection	**Standard** **Input:** Accepts standard FASTA format	Functional (GitHub) (Web server offline)	All-in-one platform; integrates feature engineering with ML pipeline	The web server is outdated/ No RNA Structural Features
iLearnPlus 2021 [35]	Web Server & Standalone GUI	Vast Sequence Descriptor Library/ Integrated Deep Learning Models	All RNA types	**High**	**Very High** Point-and-click GUI for the entire pipeline/ Automated model benchmarking	**Standard** **Input:** Accepts standard FASTA format	Functional (GitHub) (Web server offline)	State-of-the-art feature analysis & modeling suite	The web server is outdated / Requires computational resources/ No RNA Structural Features
ftrCOOL 2022 [98]	R Package (Command-line)	Nucleic acid composition; substitution matrices; k-nearest-neighbor RNA; local position-specific k-frequency; maxORF-based	All RNA types	**High** (Standalone R Package)	**Moderate** R-native for easy scripting/ No GUI/ requires programming knowledge	**Standard** **Input:** Accepts standard R data structures (vectors of sequences) or FASTA files	Active (CRAN)	Useful in R pipelines; broad descriptor library	No RNA Structural Features
PyFeat 2019 [11]	Python Toolkit (Command-line)	Z-curve; GC content; AT/GC ratio; cumulative skew; Chou’s pseudo-composition; k-gap statistics	All RNA types	**High** (Standalone Python toolkit)	**Moderate** Python-native for scripting/ No GUI/ requires programming	**Input:** Accepts sequences as strings within a Python script	Functional (GitHub)	Generates compact informative features with built-in selection	Limited feature diversity/ No GUI or web interface/ No RNA Structural Features
MathFeature 2022 [19]	Python Package (GUI & Command-line)	Numerical mapping; chaos game descriptors; Fourier transform; entropy and graph descriptors; pseudo-composition	All RNA types	**High** (Standalone Python toolkit)	**High** GUI for non-programmers/ CLI for pipeline integration	**Standard** **Input:** Accepts standard FASTA format	Functional (GitHub)	The best choice for generating features based on chaos theory, entropy, and signal processing that are not found in other tools	No RNA Structural Features
Pse-in-One 2015 [39]	Web Server & Standalone Tool	Nucleic acid composition; autocorrelation descriptors; pseudo-nucleotide composition	All RNA types	**High** (Standalone version)	**High** Intuitive web interface/ Standalone for scalability	**Standard** **Input:** Accepts standard FASTA format	Deprecated	Unified Pseudo-Component Generation	No longer accessible/ Limited RNA Structural Features
Pse-in-One 2.0 2017 [42]	Web Server & Standalone Tool	Nucleic acid composition; autocorrelation; triplet sequence-structure elements; pseudo-structure status composition; PseDPC	All RNA types	**High** (Standalone version)	**High** Intuitive web interface/ Standalone for scalability	**Standard** **Input:** Accepts standard FASTA format	Functional	Most comprehensive pseudo feature tool; includes analysis utilities	Limited RNA Structural Features
UltraPse 2017 [44]	Standalone (Command-line)	Nucleic acid composition; autocorrelation descriptors; pseudo-nucleotide composition	All RNA types	**Very High** It is a standalone command-line tool, designed specifically for high performance	**Low to Moderate** Requires compilation & scripting/ No GUI or web server	**Standard** **Input:** Accepts standard FASTA format	Functional (GitHub)	Fast & customizable for developers needing novel feature modes	Requires command-line proficiency/ No GUI or web server/ manual setup required

^a^The standalone package is explicitly designed ‘to deal with the biological sequence analysis tasks with large datasets.’ The standalone tool uses multiprocessing to reduce computation time, further enhancing its scalability for large-scale analysis. **Scalability** describes how well each tool handles increasing dataset size, ranging from strict web-server limits to high-performance standalone processing. **Ease of Use** indicates whether users can operate the tool through a simple GUI/web interface or whether programming and command-line skills are required. **Compatibility** refers to how easily each tool integrates into standard workflows, including acceptance of FASTA input, scripting interfaces, or support for Python/R environments. **Maintenance Status** notes whether the tool is functional, superseded by a newer version, or fully deprecated/offline. GUI: Graphical User Interfaces.

### A critical evaluation of feature extraction toolkits

The proliferation of software tools for RNA feature extraction presents researchers with a diverse but fragmented landscape of resources. While Table 4 provides a comprehensive inventory, a critical evaluation is necessary to guide the selection of an appropriate toolkit based on criteria such as feature comprehensiveness, computational performance, usability, and the intended analytical application.

A primary distinction is the tool’s platform, which dictates its accessibility and scalability. Web servers, such as BioTriangle and the functional Pse-in-One 2.0, offer high usability through graphical user interfaces (GUIs) that require no local installation. This makes them highly accessible for generating features from small, curated datasets. However, these platforms often have submission limits and are unsuitable for high-throughput analyses. In contrast, command-line toolkits designed as native programming packages, such as the R package ftrCOOL and the Python toolkits PyFeat and MathFeature, offer high scalability with no data-size limits. These are the superior choice for large-scale transcriptomic projects and for integration into automated, reproducible bioinformatics workflows.

Another axis of comparison is the novelty and specialization of the feature library. While most tools implement a core set of widely-used descriptors, packages like MathFeature and Nfeature are distinguished by their implementation of unique, mathematically-derived features based on concepts like chaos theory, entropy, and repeat indices, enabling novel avenues of sequence analysis. The choice of programming ecosystem is also a crucial practical consideration, with ftrCOOL serving the R community and tools like iLearnPlus and MathFeature catering to the Python environment. In summary, the selection of a feature extraction tool is a multi-factorial decision. There is no single superior tool; rather, the optimal choice involves a deliberate balance between feature scope, computational performance, accessibility, and the specific scientific objectives of the project.

## Representation learning: automated feature extraction with deep learning

In contrast to the manual design of features in traditional machine learning, representation learning offers a paradigm where informative features are learned automatically and directly from raw sequence data. This approach, powered by deep learning, has become a cornerstone of modern bioinformatics for its ability to capture complex, nonlinear patterns without prior biological assumptions [12, 13].

### From nucleotides to vectors: encoding and embedding representations

The first step in any deep learning pipeline is converting symbolic RNA sequences (A, U, C, G) into numerical form.

#### Sequence encoding

A variety of sequence encoding techniques have been developed. Common approaches include One-hot encoding [99, 100], Nucleic Acid Chemical Properties (NCP) encoding [100, 101], and Dinucleotide Physical and Chemical Properties (DPCP) encoding [102, 103]. These strategies transform RNA sequences into numerical representations using predefined rules, thereby improving the learning efficiency and predictive performance of computational models [104].

#### Embedding-based sequence representation

Inspired by natural language processing (NLP), biological sequences can be represented as collections of *k*-mers analogous to words in a sentence. Embedding models such as Word2Vec are employed to learn compact, continuous vector representations for each *k*-mer. Within this learned embedding space, *k*-mers sharing similar biological or contextual properties are positioned close to one another, effectively capturing relationships that cannot be represented by traditional one-hot encodings. These pretrained embeddings provide informative features for subsequent predictive modelling tasks [105, 106].

### Deep learning architectures as feature extractors

Once sequences are vectorized, various deep learning architectures can be used to build hierarchical feature representations.

#### Convolutional neural networks

Convolutional neural networks (CNNs) use sliding filters, known as kernels, to scan sequence representations and identify local patterns or motifs. By stacking several layers, CNNs gradually learn hierarchical features, starting from simple motifs in the early layers and progressing to more complex combinations in deeper layers [107].

#### Recurrent neural networks and long short-term memory

Recurrent neural networks (RNNs) are designed to process sequential data, making them suited for RNA sequences. They maintain an internal ‘memory’ state that captures information from previous nucleotides when processing the current one. This allows them to model long-range dependencies, which are crucial for understanding RNA structure and function. Long short-term memories (LSTMs) are an advanced form of RNN that better handle vanishing gradients, enabling them to learn dependencies over even longer sequences [108].

#### Transformers and attention mechanisms

The Transformer architecture, which has revolutionized NLP, is now making a significant impact in genomics. Its core innovation is the attention mechanism, enables the model to dynamically weigh the importance of every nucleotide in the sequence when making a prediction for a specific position. This is particularly powerful for identifying distal regulatory elements or complex structural interactions [109]. Models based on this architecture, such as BERT, can be pretrained on vast amounts of unlabelled sequence data and then fine-tuned for specific tasks. For instance, MCAMEF-BERT (multi-channel attention-based multi-encoding fusion for BERT) effectively utilizes a BERT-based model (bidirectional encoder representations from transformers) to learn discriminative features for predicting RNA N7-methylguanosine sites [110].

## Traditional feature engineering versus representation learning: a trade-off comparison

While deep learning–based representation learning has recently attracted substantial attention, traditional feature engineering continues to play an indispensable role in many RNA studies, particularly in cases where datasets are limited or interpretability is a key requirement. A clear understanding of the trade-offs between these two paradigms enables researchers to select the most appropriate approach based on their study goals, data availability, and computational resources. These paradigms differ fundamentally in their approach, requirements, and analytical outcomes. To provide a concise and practical overview, the main distinctions between traditional feature extraction and representation learning are summarized in Table 5.

**Table 5:** A comparative analysis of traditional feature extraction and representation learning

Aspect	Traditional feature extraction	Representation learning (Deep Learning)
Feature generation	Manual & Knowledge-Driven. Features are explicitly designed based on known biological or physicochemical properties.	Automatic & Data-Driven. Features are learned implicitly from raw data by the model’s layers during the training process.
Reliance on domain knowledge	Requires a deep understanding of the biological problem to select and engineer relevant features.	The model discovers patterns on its own, though domain knowledge is crucial for model architecture and result interpretation.
Interpretability	Features are often directly interpretable (e.g. ‘GC content’), supporting hypothesis generation.	Learned features are complex combinations of inputs (‘black box’). Requires *post hoc* methods for interpretation.
Predictive performance of downstream applications	Performance is capped by the quality of engineered features. Benchmarking multiple feature types and systematic feature selection or integration is recommended to optimize results.	Has the potential to capture more complex, nuanced, and context-specific relationships within input sequences when trained on sufficiently large and relevant datasets.
Data requirements	Feature extraction does not require any prior training and can be directly applied to sequences of any type and datasets of any size.	Requires either access to pretrained models in a relevant context or de novo training with substantial data.
Computational cost	Feature extraction is fast and does not require model training.	Requires training on a large dataset and is often computationally intensive.

## Discussion and conclusion

The rapid expansion of publicly available RNA sequence data has created new opportunities for computational approaches to uncover their biological roles. Despite this availability, many RNA sequences remain poorly characterized with respect to their functional and structural properties. Machine learning has emerged as a powerful framework for addressing this gap, but its success depends heavily on how effectively raw sequences are transformed into informative numerical representations. This review has provided a detailed overview of descriptor categories and feature extraction strategies for encoding RNA sequences and structures into numerical form. We have also discussed available tools and platforms that implement these methods and highlighted how the choice and diversity of feature sets can influence the predictive performance and interpretability of machine learning models in RNA-related applications.

Most prediction tasks in biological sequence analysis are framed as binary or multi-class classification problems. Numerous efficient computational approaches have been developed using machine learning algorithms to predict or analyse sequence-related characteristics solely from sequence information [97]. However, most existing machine-learning techniques, such as SVM and KNN (k-nearest neighbour), are designed to handle numerical vectors rather than raw sequences [43]. Consequently, feature extraction plays a pivotal role in converting sequences into mathematical representations that preserve their intrinsic relationship with the target variable, thereby directly influencing model performance [111]. To facilitate this process, a range of web-based servers and stand-alone software tools have been developed, enabling the extraction of diverse sequence, structural, and physicochemical features [36, 42, 43, 96]. Nevertheless, significant challenges remain. Many existing tools focus on a narrow subset of features, limiting their ability to integrate both sequence- and structure-based information in a unified framework. This limitation reduces their effectiveness for complex RNA analyses that require comprehensive feature representations.

Traditional feature extraction approaches, such as nucleic acid composition, pseudo-nucleotide composition, and autocorrelation have been widely used because of their effectiveness and simplicity in capturing sequence features. Tools like MathFeature expand on these foundations by incorporating innovative mathematical descriptors, such as chaos game theory, genomic signal processing, and entropy, which enable high accuracy across a variety of classification tasks [19]. On the other hand, some feature extraction pipelines, such as iLearnPlus [35] and the recently introduced R-based package ftrCOOL [98], have expanded the range of features by integrating physicochemical and structural descriptors alongside the aforementioned traditional approaches. ftrCOOL remarkably outperforms iLearnPlus in processing speed, making it a preferred choice for analysing large RNA datasets [98].

The primary goal of this review is to provide a systematic and accessible overview of feature extraction methods for RNA analysis. While we do not advocate a prescriptive framework, we encourage researchers to leverage the tools and techniques presented here in a modular and experimental manner. Thoughtful evaluation of different combinations, model configurations, and ensemble strategies will often yield the most reliable and generalizable results.

There is no single feature representation or machine learning model that consistently delivers optimal performance across all RNA-related tasks. The predictive value of a feature set depends on the specific biological problem, dataset composition, and the learning algorithm applied. As such, researchers are encouraged to explore a variety of machine learning models, including Support Vector Machines, Random Forests, and Gradient Boosting, while systematically evaluating feature sets both independently and in combination.

Certain features may offer strong performance in particular contexts, such as k-mer frequency for identifying RNA-binding sites or structural triplets for precursor miRNA classification. However, other features may only become informative when integrated with complementary representations. In these cases, integrative approaches and ensemble strategies, such as feature fusion, consensus modelling, and model stacking, can enhance performance by capturing diverse and nonredundant information. As demonstrated by Spooner et al. [112], integrating complementary data modalities using ensemble machine learning models can significantly improve prediction accuracy and stability compared to individual modalities or simple feature concatenation. While their study focused on clinical omics data, the underlying principle is equally relevant in RNA-based applications, where feature complementarity and diversity often influence model generalizability. This supports our view that researchers should explore combinations of feature types and models, as different biological tasks may benefit from different configurations of integrated representations and predictive frameworks.

A promising direction for future research lies in bridging the gap between the predictive power of deep learning models and the interpretability of handcrafted features. While deep architectures such as CNNs excel at capturing high-dimensional, nonlinear patterns, they often act as black boxes, offering limited biological insight. Conversely, engineered features (e.g. MFE, GC content, and specific loop counts) are grounded in domain knowledge and provide interpretable biological signals but may not capture the full complexity of RNA function on their own. To reconcile this trade-off, recent approaches have begun to explore hybrid architectures that integrate handcrafted features directly into deep learning pipelines. For example, in the EDLMFC model [113], sequence-derived and structure-informed descriptors were fused with deep neural representations, improving performance in RNA–protein interaction prediction tasks across multiple datasets. This type of architecture demonstrates how deep learning and feature engineering can be complementary rather than competing strategies.

Another important avenue is the development and adoption of benchmark datasets and shared evaluation protocols. The lack of standardized datasets and metrics has hindered direct comparisons across feature extraction methods and models. Establishing curated and diverse benchmarks that span different RNA types and prediction tasks, together with consistent metrics such as AUC, MCC, and model runtime, would provide a foundation for rigorous and reproducible evaluation.

Integrating multiple omics modalities also represents a valuable extension of current feature extraction efforts. RNA behaviour is influenced not only by sequence and structure but also by dynamic interactions with cellular factors such as proteins [114]. Combining structural and sequence-derived features with external data sources such as RBP (RNA-Binding Protein) profiles, RNA modification maps, or transcriptomic co-expression networks can yield richer and more discriminative feature spaces. This multi-modal integration aligns with emerging trends in ensemble modelling and has the potential to produce more robust classifiers that generalize well across diverse biological contexts.

Although the field has made substantial progress, further development is needed to overcome persistent challenges in accessibility, efficiency, and interoperability. Future feature extraction platforms should support a diverse array of RNA descriptors while remaining accessible to both expert users and those with limited programming experience. Emphasizing user-friendly interfaces, computational scalability, and integration capabilities will enhance reproducibility and usability. Progress will also benefit from modular, pipeline-compatible architectures that adopt standard APIs, provide well-documented libraries, and support common programming environments such as Python and R. These features will allow researchers to flexibly assemble and refine analytical workflows, fostering more adaptable and rigorous exploration in RNA bioinformatics.

**Key Points**

- This review synthesizes and categorizes over 25 distinct feature extraction methods into a structured framework (e.g. sequence-based and structure-based), creating a single point of reference in a previously fragmented field.
- This article details the computational complexity, data requirements, applications, and limitations of each feature category. This comparative framework serves as a practical guide, enabling researchers to select optimal feature extraction strategies based on specific use cases, performance trade-offs, and data availability.
- This study provides a critical evaluation of publicly available software and web servers for RNA feature extraction, distinguishing between user-friendly platforms suitable for small-scale analysis and scalable command-line toolkits essential for large datasets to guide researchers in selecting appropriate resources.
- The fundamental trade-offs between traditional manual feature engineering and emerging deep representation learning are analyzed to demonstrate that while deep learning offers automated pattern discovery for large datasets, traditional engineering remains indispensable for biological interpretability and effective modelling when dataset is small.
- The choice of feature set affects model accuracy, with evidence showing that carefully integrated feature combinations often outperform individual descriptors in RNA predictive tasks.

## Data Availability

No new datasets were generated or analyzed in this study.
